# Exploring the association between socioeconomic inequalities in chronic respiratory disease and all-cause mortality in China: findings from the China Health and Retirement Longitudinal Study

**DOI:** 10.3389/fpubh.2024.1472074

**Published:** 2025-01-07

**Authors:** Zhuo Zhang, Guoshuai Shi, Faguang Jin, Yan Zhang

**Affiliations:** ^1^School of Health Services Management, Xi’an Medical University, Xi'an, China; ^2^School of Public Health, Xi’an Medical University, Xi’an, China; ^3^Department of Respiratory and Critical Care Medicine, Tangdu Hospital, Air Force Military Medical University, Xian, China

**Keywords:** inequality, chronic respiratory disease, asthma, chronic obstructive pulmonary disease, all-cause mortality

## Abstract

**Objective:**

Research on the inequality of chronic respiratory disease (CRD) is limited, and the association between CRD and all-cause mortality is not well-established. Investigating the distribution of CRD and its associated mortality risks is essential for improving CRD conditions and developing targeted intervention measures. This study aimed to explore the relationship between inequalities in CRD and all-cause mortality in China.

**Methods:**

This study utilized nationally representative baseline data from the China Health and Retirement Longitudinal Study (CHARLS, 2011–2020, wave 1–wave 5), including a total of 14,743 subjects. The concentration index was employed to measure socioeconomic-related inequality in CRD, and the concentration index decomposition method was used to describe its influencing factors. Cox proportional hazards regression model was employed to examine the association between CRD and all-cause mortality.

**Results:**

The prevalence of CRD was 11.79% (95% CI: 10.98, 12.66) in China. The concentration index for CRD was −0.050 (95% CI: −0.075, −0.026), indicating a certain degree of inequality in its prevalence. Chronic lung disease (concentration index = −0.046, 95% CI: −0.073, −0.019), asthma (concentration index = −0.102, 95% CI: −0.148, −0.056), and asthma-chronic obstructive pulmonary disease overlap syndrome (concentration index = −0.114, 95% CI: −0.173, −0.055) also exhibited a pro-poor distribution. The decomposition analysis of the concentration index for CRD revealed that age, education level, and economic status played substantial roles in contributing to the observed inequality. Additionally, Cox regression analysis showed that participants with CRD had an increased risk of all-cause mortality (HR = 1.49, 95% CI: 1.34, 1.65).

**Conclusion:**

Inequalities exists in CRDs in China, with the prevalence of these diseases primarily concentrated among economically disadvantaged groups. Additionally, CRD increases the risk of all-cause mortality. Addressing the root causes of economic inequalities and enhancing the educational attainment of individuals with low socioeconomic status can help improve the situation.

## Introduction

Chronic respiratory disease (CRD), primarily chronic lung diseases, are a significant category of non-communicable diseases that pose a serious threat to health (1). Globally, the prevalence of CRD stands at 7.1%, affecting an estimated 545 million people. Ranked as the third leading cause of death worldwide, following cardiovascular disease and cancer, this group of diseases is responsible for approximately 4 million deaths (2, 3). In China, CRD are also widespread, securing the fourth position among causes of death in 2019 and accounting for 10.6% of total deaths in the country (4). Beyond the threat to life, CRD can result in diminished physical function, leading to disability and increased medical costs, imposing a substantial burden on families and society (5, 6).

Asthma and chronic obstructive pulmonary disease (COPD) are prevalent CRD (7). Asthma is characterized by chronic airway inflammation, leading to recurrent wheezing, shortness of breath, coughing, and chest tightness (8). Chronic obstructive pulmonary disease, encompassing chronic bronchitis and emphysema, is characterized by persistent airflow restriction and corresponding respiratory symptoms (9). Global prevalence data indicate that asthma affects approximately 334 million people (10), while COPD affects over 200 million people, with about 65 million experiencing moderate or severe disease, making it the third leading cause of death worldwide. Notably, more than three-quarters of individuals with COPD reside in low- and middle-income countries (11, 12). Asthma and COPD also impose a significant health burden in China. Data from the China Lung Health Study reveal that the prevalence of asthma in individuals aged 20 and above in China is 4.2%, with approximately 45.7 million patients (13). As for COPD, the prevalence is 13.7% in individuals over 40 years of age, affecting nearly 100 million people nationwide (14). Studies have demonstrated the existence of asthma-chronic obstructive pulmonary disease overlap syndrome (ACOS), although there is no consistent agreement on the diagnostic criteria for this condition (15). Moreover, the clinical characteristics of this syndrome are complex, resulting in worse health status, increased treatment difficulty, and a significantly elevated risk of poor patient prognosis (16). Therefore, in-depth research and more effective management strategies for CRD are essential to reduce their burden.

Socioeconomic status serves as a comprehensive indicator of an individual’s economic and social standing, used to gauge their social status. Generally, a higher socioeconomic status tends to be positively correlated with better health. Numerous studies have identified an association between socioeconomic status and the prevalence of chronic diseases (17). One study highlighted socioeconomic inequalities among patients with various fatal chronic diseases (18). Moreover, studies conducted on the Slovenian population with chronic diseases have revealed a significantly higher incidence of chronic conditions among individuals with lower socioeconomic and employment status (19). In measuring health inequities resulting from socioeconomic factors, the concentration index is widely employed to assess health equity, and its reliability has been well validated in previous studies (20). Additionally, previous studies have shown that CRD may affect all-cause mortality, and a meta-analysis of cohort studies indicated that patients with asthma had an increased risk of all-cause mortality (21). A large national cohort study also found that patients with COPD had a significantly higher risk of all-cause death compared to those without COPD (22).

Numerous studies have highlighted a robust association between socioeconomic status and CRD. However, different studies have presented conflicting results (18, 23). Additionally, despite previous research analyzing the relationship between CRD and all-cause mortality, the literature on the relationship between CRD inequality and all-cause mortality in low- and middle-income countries, especially in China, remains limited. Furthermore, even within large cohorts, results are not entirely consistent, indicating the need for further research. To fill this gap, we used data from the China Health and Retirement Longitudinal Study (CHARLS) to analyze the relationship between CRD and all-cause mortality among Chinese adults and explore the inequitable status of CRD.

## Materials and methods

### Data sources and study population

The study utilized 2011–2012 baseline data (wave 1) and follow-up data (wave2-wave5) from the CHARLS. Initiated by the National School of Development at Peking University, CHARLS conducted a national baseline survey in 2011, followed by four subsequent visits in 2013, 2015, 2018, and 2020. This study adopted a multi-stage stratified probability proportional scale sampling method to sample residents over 45 years old in 150 counties and 450 communities/villages across 28 provinces (autonomous regions and municipalities directly under the Central Government) in China. The survey encompassed two main components: a household questionnaire and a physical examination survey designed to collect detailed information from the respondents. The purpose of data collection was to provide fundamental insights into China’s aging population, supporting the formulation of more effective policy programs aimed at improving the living conditions of the older adult population. A scientific sampling method was employed to ensure that the large sample of older adult people was nationally representative. The information on chronic lung diseases and asthma was obtained through a questionnaire survey, and any missing samples were deleted to ensure the data’s integrity and accuracy. The CHARLS project received ethics approval from the Peking University Ethical Review Committee (IRB00001052-11015), and subjects provided written informed consent before the investigation commenced, ensuring the ethical compliance of the study. Moreover, the present analysis received approval from Xi’an Medical University Medical Ethics Review Committee (XYLS2023077).

In this study, individuals with missing CRD information (n = 278), missing economic status information (n = 293), those under 45 years of age (n = 489), and those lost to follow-up (n = 1,905) were excluded. Finally, 14,743 participants were included in the analysis.

### Study variables

Chronic respiratory diseases were the main variable in this study, encompassing chronic lung disease and asthma, as obtained from CHALRS questionnaires. The assessment of chronic lung disease involved the question, ‘Have you been diagnosed with chronic lung diseases, such as chronic bronchitis, emphysema (excluding tumors, or cancer) by a doctor?’ Asthma was determined through the question, ‘Have you been diagnosed with asthma by a doctor?’ ACOS (asthma-chronic obstructive pulmonary disease overlap syndrome) was not specifically investigated in this study. Since COPD is a significant component of chronic lung disease (24), and there is no uniform definition for ACOS, the comorbidities of chronic lung disease and asthma investigated in this study were considered as ACOS.

The primary outcome of the study was all-cause mortality. Mortality information was collected from the wave 2 to wave 5, with only wave 2 providing an exact date of death. If participants survived during the follow-up period, their survival time was the interval between the two surveys. If they died, the survival time was the interval from the date of wave 1 to the date of death of the participant, or from the date of wave 1 to the median time of the wave with recorded death.

### Covariance

The covariates included in this study encompassed basic information such as age (45–59, 60–74, ≥75), gender (male, female), educational level (illiterate, primary school, secondary/high school, university or above), and marriage status (never married, married, others). Lifestyle factors, including smoking (no, yes), drinking (never, occasionally, regularly), BMI (0–23.9, 24–27.9, ≥28), basic health insurance (no, yes), and preventative health service utilization (no, yes), were also considered. BMI was calculated using the weight/height squared formula, and alcohol consumption was categorized as never, occasionally (defined as less than one drink per month), and regularly (defined as more than one drink per month).

### Statistical analysis

First, the concentration index was utilized to assess the inequality of CRD, chronic lung disease, asthma, and the ACOS. The economic status indicator employed was the IM_PCE from the “Constructed Expenditure, Income, and Wealth Database” released by CHARLS in 2017. Economic status was categorized into five groups based on quartiles. To further validate the results, we conducted a stratified analysis of the inequality of the CRD based on age and sex. Data were extrapolated by applying the CHARLS sampling weight (ind_weight_ad2) to estimate the prevalence of CRD among Chinese adults aged 45 years and older. Secondly, we described the basic characteristics of the study population. Continuous data conforming to normal distribution were presented as means and standard deviations, while frequencies and percentages were used for categorical data. Thirdly, Concentration index decomposition was employed to describe the contribution of each influencing factor to the inequity. Finally, a Cox proportional hazards regression model was employed to examine the association between CRD and all-cause mortality. Additionally, a stratified analysis was performed according to sociodemographic characteristics. Stata 16.0 was used for data collation and analysis, with *p* < 0.05 considered statistically significant.

## Results

### Concentration index

**Figure 1:**
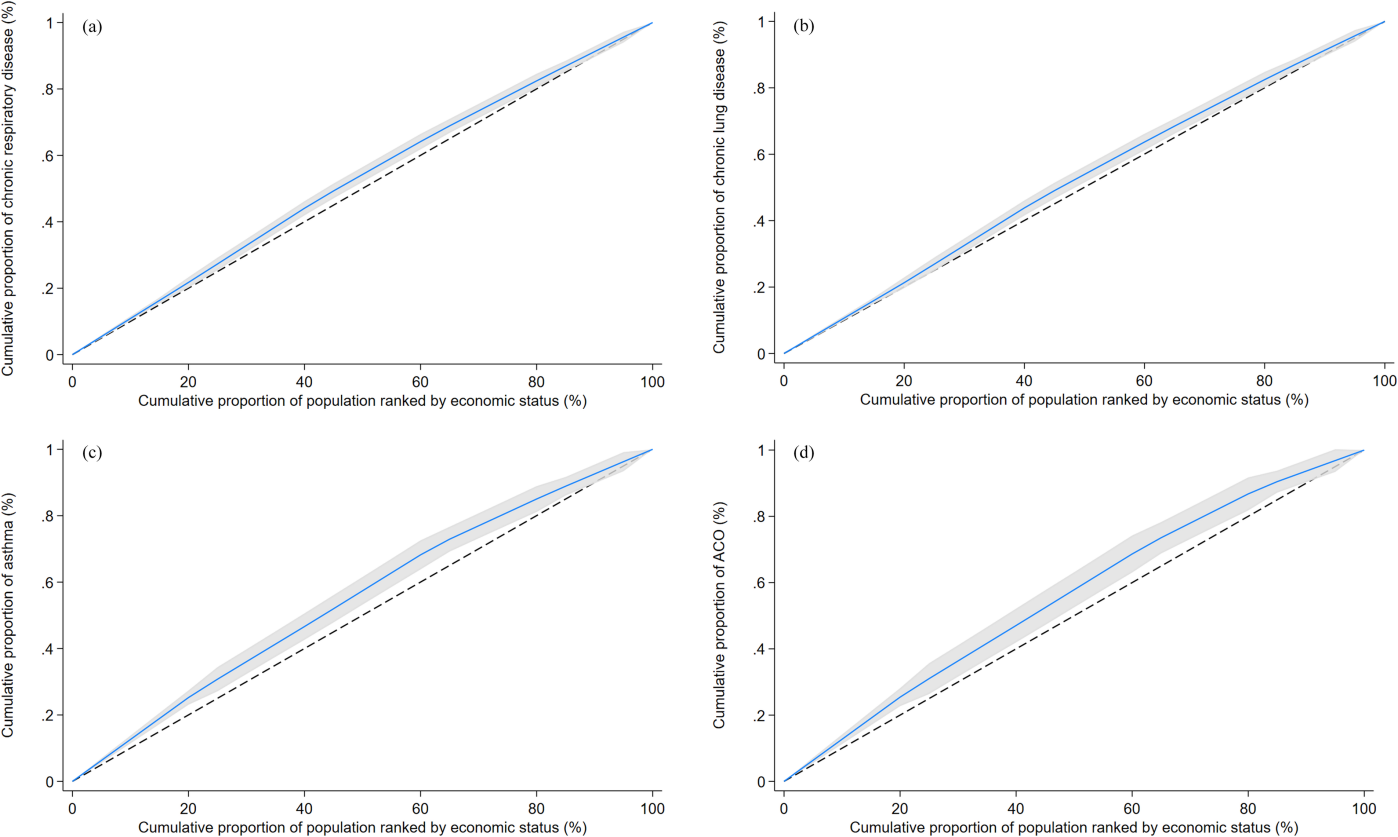
Concentration curves for chronic respiratory disease **(A)**, chronic lung disease **(B)**, asthma **(C)** and ACOS **(D)**. The dotted line surrounding the curve represents the 95% CI. ACOS, asthma-chronic obstructive pulmonary disease overlap syndrome.

The prevalence of CRD, chronic lung disease, asthma, and ACOS were 11.79% (95% CI: 10.98, 12.66), 10.42% (95% CI: 9.65, 11.24), 3.72% (95% CI: 3.29, 4.20), and 2.34% (95% CI: 2.03, 2.71), respectively. The concentration indexes for CRD, chronic lung disease, asthma, and ACOS were −0.050 (95% CI: −0.075, −0.026), −0.046 (95% CI: −0.073, −0.019), −0.102 (95% CI: −0.148, −0.056), and −0.114 (95% CI: −0.173, −0.055) (Figure 1; Supplementary Table S1). The results indicated a certain degree of inequality in the prevalence of CRD, chronic lung disease, asthma, and ACOS, with the prevalence of these diseases mainly concentrated in the population with poor economic status.

### Stratified analysis

To further demonstrate the inequity in the prevalence of CRD in different populations, we conducted a subgroup analysis. We found that several diseases were more prevalent among individuals with lower socioeconomic status in both men and participants aged <60, consistent with the results of the main study. However, ACOS did not show significant inequities in women. No inequities were observed in several diseases among participants older than 60 years (Table 1).

**Table 1:** Concentration index for chronic respiratory disease, chronic lung disease, asthma and ACOS by gender and age.

	Concentration index	95% CI	*p*
Male			
Chronic respiratory disease	−0.046	−0.079, −0.013	0.006
Chronic lung disease	−0.048	−0.083, −0.013	0.008
Asthma	−0.122	−0.183, −0.061	<0.001
ACOS	−0.168	−0.244, −0.093	<0.001
Female			
Chronic respiratory disease	−0.058	−0.096, −0.020	0.003
Chronic lung disease	−0.045	−0.087, −0.004	0.031
Asthma	−0.078	−0.148, −0.007	0.031
ACOS	−0.033	−0.127, 0.061	0.489
<60, years			
Chronic respiratory disease	−0.073	−0.113, −0.033	<0.001
Chronic lung disease	−0.076	−0.118, −0.033	0.001
Asthma	−0.093	−0.171, −0.015	0.020
ACOS	−0.120	−0.222, −0.019	0.020
**≥60, years**			
Chronic respiratory disease	0.005	−0.026, 0.036	0.758
Chronic lung disease	0.013	−0.021, 0.047	0.462
Asthma	−0.058	−0.115, 0.001	0.046
ACOS	−0.062	−0.135, 0.010	0.089

ACOS, asthma-chronic obstructive pulmonary disease overlap syndrome.

### Sociodemographic characteristics of the subjects

A total of 14,743 subjects were enrolled in this study, with an average age of 59.72 ± 9.83, comprising 7,232 males (49.05%). The majority of participants had an education level of high school and below (98.22%), and 86.96% were married. Additionally, 32.33% were smokers, 41.96% were drinkers, and 31.68% were overweight or obese. Moreover, 91.06% had basic medical insurance, and 19.85% had utilized basic public health services in the previous month (Table 2).

**Table 2:** Sociodemographic characteristics of subjects aged 45 years and older.

Variables	Categories	*N* (%)
Age group, (years)	45–59	8,289 (56.22)
	60–74	5,185 (35.17)
	≥75	1,269 (8.61)
Gender	Male	7,232 (49.05)
	Female	7,511 (50.95)
Educational level	Illiterate	4,272 (28.98)
	Primary school	5,874 (39.84)
	Secondary/high school	4,334 (29.40)
	University or above	263 (1.78)
Marriage status	Never married	140 (0.95)
	Married	12,820 (86.96)
	Others	1783 (12.09)
Smoking	No	9,976 (67.67)
	Yes	4,767 (32.33)
Drinking	Never	8,553 (58.05)
	Occasionally	1,514 (10.28)
	Regularly	4,667 (31.68)
BMI (kg/m^2^)	0–23.9	10,073 (68.32)
	24–27.9	3,351 (22.73)
	≥28	1,319 (8.95)
Basic health insurance	No	1,318 (8.94)
	Yes	13,425 (91.06)
Preventative health service	No	11,816 (80.15)
	Yes	2,927 (19.85)
Economic status	0–20%	3,127 (21.21)
	20–40%	3,077 (20.87)
	40–60%	3,006 (20.39)
	60–80%	2,908 (19.72)
	80–100%	2,625 (17.81)

BMI, body mass index.

### Concentration index decomposition

The decomposition analysis of the concentration index for CRD revealed that age, education level, and economic status played substantial roles in contributing to the inequality observed in these diseases. Older age, lower educational attainment, and poorer economic status were identified as factors that elevate the inequality associated with CRD. Additionally, the study found that other factors such as marital status, smoking, and higher BMI also contributed to an increase in inequality related to CRD (Table 3).

**Table 3:** Decomposition analysis on the inequality of chronic respiratory diseases.

Variables	Categories	Elasticity	Concentration index	Contribution	Contribution rate (%)
Age group, (years)	60–74	1.633	−0.077	−0.126	37.68
	≥75	0.551	−0.142	−0.078	
Gender	Female	−1.567	−0.004	0.007	−1.28
Educational level	Illiterate	1.604	−0.188	−0.302	45.25
	Primary school	2.025	−0.041	−0.082	
	Secondary/high school	0.695	0.200	0.139	
Marriage status	Never married	0.012	−0.174	−0.002	1.71
	Others	0.156	−0.046	−0.007	
Smoking	Yes	0.226	−0.014	−0.003	0.58
Drinking	No	−0.398	−0.007	0.003	−0.50
	Sometimes	−0.041	0.005	0.000	
BMI (kg/m^2^)	24–27.9	−0.279	0.059	−0.016	3.00
	≥28	0.003	0.063	0	
Basic health insurance	Yes	−1.386	−0.008	0.011	−2.11
Preventative health service	Yes	1.09	0.014	0.015	−2.79
Economic status	0–20%	0.01	−0.788	−0.008	18.47
	20–40%	0.213	−0.367	−0.078	
	40–60%	0.06	0.046	0.003	
	60–80%	−0.037	0.447	−0.016	

BMI, body mass index.

### Association of chronic respiratory disease with risks of all-cause mortality

**Figure 2:**
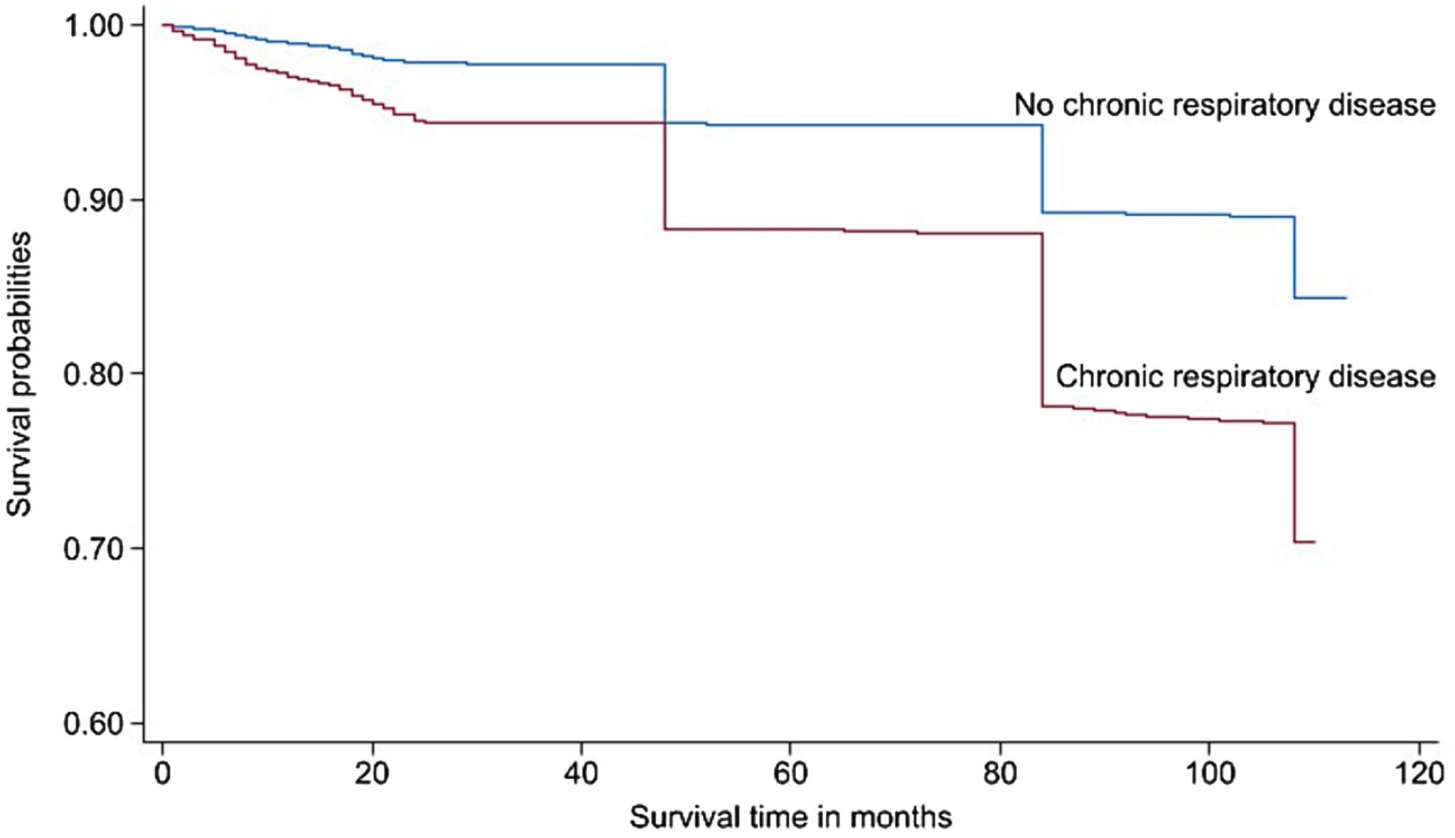
Kaplan–Meier curve showing the cumulative risk of all-cause mortality by chronic respiratory disease status.

Over the approximately 10-years follow-up period, 2,368 deaths were reported, resulting in a mortality rate of 15.76% (95% CI: 15.14, 16.41). Subjects with CRD had a higher all-cause mortality rate than those without CRD (Log-rank test, *p* < 0.05) (Figure 2). After adjusting for all covariates, Cox regression analysis revealed that participants with CRD had an increased risk of all-cause mortality (HR = 1.49, 95% CI: 1.34, 1.65) (Supplementary Table S2). The analysis of specific types of CRD revealed that chronic lung disease, asthma and ACOS were significantly associated with an increased risk of all-cause mortality (Supplementary Table S2). Additionally, stratified analyses based on sociodemographic characteristics showed that while the results for each subgroup varied slightly from the overall findings, there was a consistent trend indicating that CRD was associated with a higher risk of all-cause mortality across different groups (Supplementary Figure S1).

## Discussion

We used nationally representative data from the CHARLS baseline survey to examine the socioeconomic inequalities associated with CRD. Our study identified inequalities in CRD and its specific types (chronic lung disease, asthma, ACOS), predominantly favoring individuals of lower economic status. Age, education level and economic status were identified as significant factors contributing to these inequalities. Additionally, our findings indicated that CRD was associated with an increased risk of all-cause mortality. These results highlight the current inequitable burden of CRD and its potential implications for mortality in China.

Previous studies had revealed the inequity present in common chronic diseases, such as hypertension and diabetes (25). This study aligned with those findings, indicating a similar inequality in CRD. Adam et al., utilizing multiple rounds of cross-sectional data from the United States, demonstrated the existence and worsening of socioeconomic inequality in lung health over time (26). Furthermore, socioeconomic status emerged as a potential independent determinant of lung health. On a global scale, inequalities in CRD were also apparent. Haifeng Li et al., drawing on data from the global disease burden, identified a concentration of disability-adjusted life years associated with COPD in less developed countries (27). The socioeconomic status-driven inequality in CRD may be linked to insufficient healthcare programs and inadequate tobacco consumption control (27, 28). These findings underscored the importance of health system reform, emphasizing the need to direct more attention to vulnerable groups based on economic and social status. Additionally, CRD served as predictors of all-cause mortality (29, 30), suggesting that the income-based gap in CRD identified in our study may contribute to life expectancy inequality in China.

This study highlighted the significant impact of age, education, and economic status on chronic respiratory inequality. It was widely recognized that older adults often encounter challenges such as physical decline and a weakened immune system, increasing their susceptibility to CRD (31). A potential remedy could involve providing increased access to free basic public health services and allocating more resources to medicare drugs related to respiratory health (32). In terms of age and education, economic status emerged as a key modifiable risk factor. Previous research had identified low economic status as a risk factor for CRD (27, 28), exposing individuals to additional risk factors like poor dietary habits and increased occupational dust exposure (33). Conversely, individuals with higher economic status were more likely to leverage medical technology for health maintenance, receive health advice, actively engage in disease screenings (25, 26), and ultimately reduce the risk of CRD.

Respiratory diseases are a major cause of death worldwide and rank as the third leading cause of death globally (29). In 2017, CRDs were responsible for nearly 4 million deaths, marking an 18 percent increase since 1990 (2). Population-based studies have also reported that CRD increases the risk of all-cause mortality. Bin Zhang et al. summarized the findings of 30 cohort studies, revealing that patients with asthma had a higher all-cause mortality rate (RR = 1.38, 95% CI: 1.07, 1.77) (21). A meta-analysis pooling the results of 12 studies found that even mild COPD was associated with increased all-cause mortality (34). Additionally, a study using data from the National Health and Nutrition Examination Survey found that patients with ACO had a higher risk of all-cause death compared to individuals with asthma only, COPD only, non-asthma/COPD, or non-ACO (35). These findings are consistent with our study. The increased risk of all-cause mortality in patients with CRD not only from the disease itself but also from chronic inflammation, airway limitation, and comorbidities due to medication (e.g., osteoporosis, pneumonia, depression) (36, 37). Implementing appropriate management strategies and interventions is crucial to reducing this risk.

This study analyzed the inequality of CRD and its risk of all-cause mortality using nationally representative data, providing a crucial foundation for the control of such diseases. However, the article has several limitations. Firstly, the study did not categorize the collection of chronic lung diseases, making it challenging to determine the percentage of COPD. Consequently, chronic lung diseases were used to represent COPD, introducing a certain degree of bias. Nevertheless, considering that COPD is the primary chronic lung disease, this bias is likely minimal. Secondly, the prevalence of CRD relies on self-reporting, which may introduce bias, given the low awareness rates of diseases such as asthma and COPD in China (38, 39). Additionally, due to age limitations in the survey, we were unable to estimate the inequality of CRD in individuals over 75 years of age. Finally, this study employs data from 2011, potentially introducing bias compared to the current reality of CRD inequality.

## Conclusion

There is inequality in CRD in China, primarily affecting economically disadvantaged groups, and CRD increased the risk of all-cause death. Age, education level, and economic factors significantly contribute to this inequality. Policymakers and researchers should prioritize the needs of individuals with low socioeconomic status when developing strategies to prevent CRD. Addressing the root causes of income inequality and improving educational attainment among these individuals can help mitigate the issue.

## Data Availability

Publicly available datasets were analyzed in this study. This data can be found at: http://charls.pku.edu.cn/.

## References

[1] Laxmi V. Clarification of non communicable diseases their types and risk factor and some preventive action to reduce the chronic diseases. Pharma Innov J. (2022) 11:1896–907.

[2] Soriano JB Kendrick PJ Paulson KR Gupta V Abrams EM Adedoyin RA. Prevalence and attributable health burden of chronic respiratory diseases, 1990–2017: A systematic analysis for the global burden of disease study 2017. Lancet Respir Med. (2020) 8:585–96. doi: 10.1016/S2213-2600(20)30105-3, PMID: 32526187 PMC7284317

[3] Vos T Lim SS Abbafati C Abbas KM Abbasi M Abbasifard M. Global burden of 369 diseases and injuries in 204 countries and territories, 1990–2019: A systematic analysis for the global burden of disease study 2019. Lancet. (2020) 396:1204–22. doi: 10.1016/S0140-6736(20)30925-9, PMID: 33069326 PMC7567026

[4] Long Z Liu W Qi J-L Liu Y-N Liu J-M You J-L. Mortality trend of chronic respiratory diseases in China, 1990-2019. Zhonghua Liu Xing Bing Xue Za Zhi. (2022) 43:14–21. doi: 10.3760/cma.j.cn112338-20210601-00443 35130647

[5] Booth S Johnson MJ. Improving the quality of life of people with advanced respiratory disease and severe breathlessness. Breathe. (2019) 15:198–215. doi: 10.1183/20734735.0200-2019 31508158 PMC6717608

[6] Beran D Zar HJ Perrin C Menezes AM Burney P. Burden of asthma and chronic obstructive pulmonary disease and access to essential medicines in low-income and middle-income countries. Lancet Respir Med. (2015) 3:159–70. doi: 10.1016/S2213-2600(15)00004-1, PMID: 25680912

[7] Labaki WW Han MK. Chronic respiratory diseases: A global view. Lancet Respir Med. (2020) 8:531–3. doi: 10.1016/S2213-2600(20)30157-0 32526184 PMC8034823

[8] Cevhertas L Ogulur I Maurer DJ Burla D Ding M Jansen K. Advances and recent developments in asthma in 2020. Allergy. (2020) 75:3124–46. doi: 10.1111/all.14607 32997808

[9] Martinez FJ Han MK Allinson JP Barr RG Boucher RC Calverley PM. At the root: defining and halting progression of early chronic obstructive pulmonary disease. Am J Respir Crit Care Med. (2018) 197:1540–51. doi: 10.1164/rccm.201710-2028PP, PMID: 29406779 PMC6006401

[10] Enilari O Sinha S. The global impact of asthma in adult populations. Ann Glob Health. (2019) 85:1–7. doi: 10.5334/aogh.2412 PMC7052341 30741503

[11] Ruvuna L Sood A. Epidemiology of chronic obstructive pulmonary disease. Clin Chest Med. (2020) 41:315–27. doi: 10.1016/j.ccm.2020.05.002, PMID: 32800187

[12] Adeloye D Song P Zhu Y Campbell H Sheikh A Rudan I. Global, regional, and national prevalence of, and risk factors for, chronic obstructive pulmonary disease (Copd) in 2019: A systematic review and modelling analysis. Lancet Respir Med. (2022) 10:447–58. doi: 10.1016/S2213-2600(21)00511-7, PMID: 35279265 PMC9050565

[13] Huang K Yang T Xu J Yang L Zhao J Zhang X. Prevalence, risk factors, and management of asthma in China: A National Cross-Sectional Study. Lancet. (2019) 394:407–18. doi: 10.1016/S0140-6736(19)31147-X 31230828

[14] Wang C Xu J Yang L Xu Y Zhang X Bai C. Prevalence and risk factors of chronic obstructive pulmonary disease in China (the China pulmonary health [Cph] study): A National Cross-Sectional Study. Lancet. (2018) 391:1706–17. doi: 10.1016/S0140-6736(18)30841-9 29650248

[15] Postma DS Rabe KF. The asthma–COPD overlap syndrome. N Engl J Med. (2015) 373:1241–9. doi: 10.1056/NEJMra1411863 26398072

[16] Maselli DJ Hanania NA. Management of Asthma Copd Overlap. Ann Allergy Asthma Immunol. (2019) 123:335–44. doi: 10.1016/j.anai.2019.07.021 31376487

[17] Mair FS Jani BD. Emerging trends and future research on the role of socioeconomic status in chronic illness and multimorbidity. Lancet Public Health. (2020) 5:e128–9. doi: 10.1016/S2468-2667(20)30001-3, PMID: 32007133

[18] Sommer I Griebler U Mahlknecht P Thaler K Bouskill K Gartlehner G. Socioeconomic inequalities in non-communicable diseases and their risk factors: An overview of systematic reviews. BMC Public Health. (2015) 15:1–12. doi: 10.1186/s12889-015-2227-y 26385563 PMC4575459

[19] Softič N Smogavec M Klemenc-Ketiš Z Kersnik J. Prevalence of chronic diseases among adult Slovene population. Slovenian J Public Health. (2011) 50:185–90. doi: 10.2478/v10152-010-0043-4

[20] Kakwani N Wagstaff A Van Doorslaer E. Socioeconomic inequalities in health: measurement, computation, and statistical inference. J Econ. (1997) 77:87–103. doi: 10.1016/S0304-4076(96)01807-6

[21] Zhang B Li Z-F An Z-Y Zhang L Wang J-Y Hao M-D. Association between asthma and all-cause mortality and cardiovascular disease morbidity and mortality: A Meta-analysis of cohort studies. Front Cardiovasc Med. (2022) 9:861798. doi: 10.3389/fcvm.2022.861798, PMID: 35369308 PMC8968068

[22] Park HY Kang D Lee H Shin SH Kang M Kong S. Impact of chronic obstructive pulmonary disease on mortality: A large National Cohort Study. Respirology. (2020) 25:726–34. doi: 10.1111/resp.13678, PMID: 31426128

[23] Williams DR Mohammed SA Leavell J Collins C. Race, socioeconomic status, and health: complexities, ongoing challenges, and research opportunities. Ann N Y Acad Sci. (2010) 1186:69–101. doi: 10.1111/j.1749-6632.2009.05339.x 20201869 PMC3442603

[24] Song P Zha M Xia W Zeng C Zhu Y. Asthma–chronic obstructive pulmonary disease overlap in China: prevalence, associated factors and comorbidities in middle-aged and older adults. Curr Med Res Opin. (2020) 36:667–75. doi: 10.1080/03007995.2020.1722082, PMID: 31992091

[25] Mira R Newton T Sabbah W. Inequalities in the Progress of multiple chronic conditions: A systematic review of longitudinal studies. PLoS One. (2022) 17:e0263357. doi: 10.1371/journal.pone.0263357, PMID: 35113920 PMC8812855

[26] Gaffney AW Himmelstein DU Christiani DC Woolhandler S. Socioeconomic inequality in respiratory health in the US from 1959 to 2018. JAMA Intern Med. (2021) 181:968–76. doi: 10.1001/jamainternmed.2021.2441, PMID: 34047754 PMC8261605

[27] Li H Liang H Wei L Shi D Su X Li F. Health inequality in the global burden of chronic obstructive pulmonary disease: findings from the global burden of disease study 2019. Int J Chron Obstruct Pulmon Dis. (2022) 17:1695–702. doi: 10.2147/COPD.S369120, PMID: 35923358 PMC9342709

[28] Sahni S Talwar A Khanijo S Talwar A. Socioeconomic status and its relationship to chronic respiratory disease. Adv Respir Med. (2017) 85:97–108. doi: 10.5603/ARM.2017.0016 28440535

[29] Li X Cao X Guo M Xie M Liu X. Trends and risk factors of mortality and disability adjusted life years for chronic respiratory diseases from 1990 to 2017: systematic analysis for the global burden of disease study 2017. BMJ. (2020):368. doi: 10.1136/bmj.m234 PMC7190065 32075787

[30] Salciccioli JD Marshall DC Shalhoub J Maruthappu M De Carlo G Chung KF. Respiratory disease mortality in the United Kingdom compared with Eu15+ countries in 1985-2015: observational study. BMJ. (2018):k4680. doi: 10.1136/bmj.k4680 30487157 PMC6259045

[31] Cho SJ Stout-Delgado HW. Aging and lung disease. Annu Rev Physiol. (2020) 82:433–59. doi: 10.1146/annurev-physiol-021119-034610 31730381 PMC7998901

[32] Adler NE Glymour MM Fielding J. Addressing social determinants of health and health inequalities. JAMA. (2016) 316:1641–2. doi: 10.1001/jama.2016.14058 27669456

[33] Yin P Zhang M Li Y Jiang Y Zhao W. Prevalence of Copd and its association with socioeconomic status in China: findings from China chronic disease risk factor surveillance 2007. BMC Public Health. (2011) 11:1–8. doi: 10.1186/1471-2458-11-586 21781320 PMC3152537

[34] Weifeng Z Jie O Fan W Huanuan F Yuyan H Haiqing L. Association of mild chronic obstructive pulmonary disease with all-cause mortality: A systematic review and meta-analysis. Pulmonology. (2023):S2531-0437(23)00165-4. doi: 10.1016/j.pulmoe.2023.09.002 38093693

[35] Meng Z An C. Epidemiological characteristics of asthma-Copd overlap, its association with all-cause mortality, and the mediating role of depressive symptoms: evidence from Nhanes 2005-2018. BMC Public Health. (2024) 24. doi: 10.1186/s12889-024-18911-1, PMID: 38807148 PMC11134654

[36] Gershon AS Wang C Guan J To T. Burden of comorbidity in individuals with asthma. Thorax. (2010) 65:612–8. doi: 10.1136/thx.2009.131078 20627918

[37] Odegaard AO Jacobs DR Sanchez OA Goff DC Reiner AP Gross MD. Oxidative stress, inflammation, endothelial dysfunction and incidence of type 2 diabetes. Cardiovasc Diabetol. (2016) 15:1–12. doi: 10.1186/s12933-016-0369-6 27013319 PMC4806507

[38] Quan Z Yan G Wang Z Li Y Zhang J Yang T. Current status and preventive strategies of chronic obstructive pulmonary disease in China: A literature review. J Thorac Dis. (2021) 13:3865–77. doi: 10.21037/jtd-20-2051, PMID: 34277076 PMC8264680

[39] Cong S Yao J Fan J Wang N Wang B Bao H. Analysis on awareness of chronic obstructive pulmonary disease (Copd) status and related knowledge in patients with Copd in China, 2014-2015. Zhonghua Liu Xing Bing Xue Za Zhi. (2020) 41:1034–40. doi: 10.3760/cma.j.cn112338-20200206-00074, PMID: 32741166

