# Supplementary material for: Exploring the association between socioeconomic inequalities in chronic respiratory disease and all-cause mortality in China: findings from the China Health and Retirement Longitudinal Study
Source: Front Public Health. 2025 Jan 7;12:1472074. doi: 10.3389/fpubh.2024.1472074 (PMC11746896; doi:10.3389/fpubh.2024.1472074)
Supplement: Supplementary file 1 [file Data_Sheet_1.docx]

**Supplementary Table 1.** Concentration index for chronic respiratory disease, chronic lung disease asthma and ACOS

|  | Concentration index | 95%CI | P |
| --- | --- | --- | --- |
| **Chronic respiratory disease** | -0.050 | -0.075, -0.026 | <0.001 |
| chronic lung disease | -0.046 | -0.073, - 0.019 | 0.001 |
| asthma | -0.102 | -0.148, -0.056 | <0.001 |
| ACOS | -0.114 | -0.173, -0.055 | <0.001 |

ACOS: asthma-chronic obstructive pulmonary disease overlap syndrome

**Supplementary Table 2.** Association between chronic respiratory disease and its specific types

and the risk of all-cause mortality

|  | HR | 95%CI | P |
| --- | --- | --- | --- |
| **Chronic respiratory disease** | 1.49 | 1.34, 1.65 | <0.001 |
| chronic lung disease | 1.52 | 1.37, 1.69 | <0.001 |
| asthma | 1.54 | 1.32, 1.80 | <0.001 |
| ACOS | 1.75 | 1.47, 2.10 | <0.001 |

ACOS: asthma-chronic obstructive pulmonary disease overlap syndrome


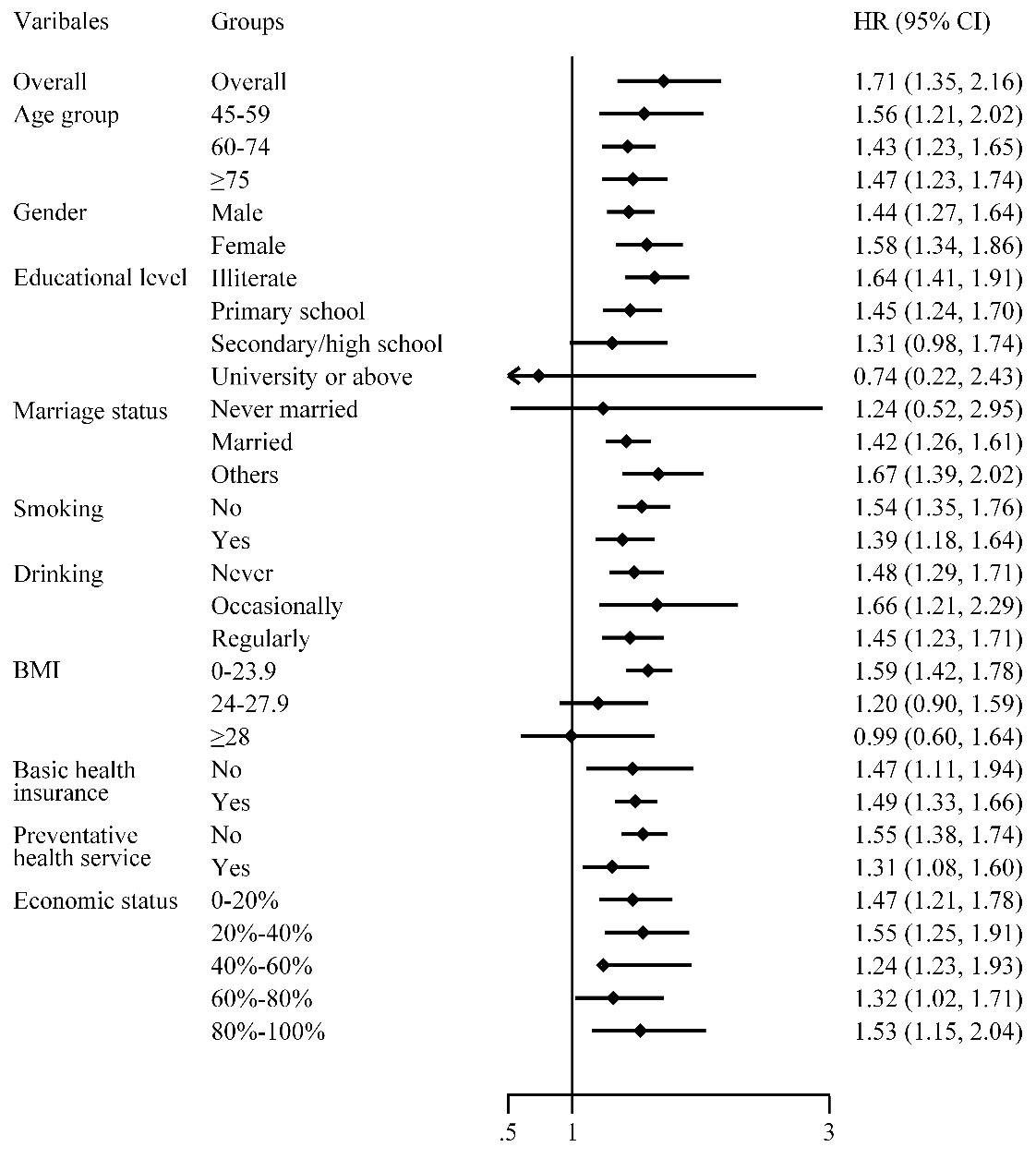


**Supplementary Figure 1.** Association between chronic respiratory disease and the risk of all-cause mortality according to sociodemographic characteristics. Adjustments were made for age, gender, education level, marital status, smoking, drinking, BMI, basic health insurance, preventative health services and economic status, in addition to the subgroup variables.
